# Exploring the influence of cultural participation on the subjective well-being of victims in Mexico

**DOI:** 10.3389/fpsyg.2022.1082216

**Published:** 2023-01-10

**Authors:** Javier Reyes-Martínez, Oscar A. Martínez-Martínez, Margaret Lombe, María Piñeros-Leaño

**Affiliations:** ^1^El Colegio de México, Ciudad de México, Mexico; ^2^Departamento de Ciencias Sociales y Políticas, Universidad Iberoamericana, Ciudad de México, Mexico; ^3^School of Social Work, Boston University, Boston, MA, United States; ^4^Social Work School, Boston College, Chestnut Hill, MA, United States

**Keywords:** subjective well-being, cultural participation, victimization, victims, Mexico

## Abstract

**Introduction:**

Considering the increasing incidence of crime in Mexico, it is necessary to understand the strategies that individuals utilize in response to victimization and the effects of this on their subjective well-being.

**Methods:**

A generalized structural equation modeling (GSEM) analysis with data from the 2012 Self-reported Well-Being Survey (BIARE, *n* = 10,654); dependent variables: subjective well-being (i.e., cognitive well-being and affective balance); independent variables: self-reported victimization (i.e., by domestic violence, community violence, and structural violence) and cultural participation (i.e., cultural attendance, engagement, and consumption).

**Results:**

Results show an overall positive and statistical influence of the cultural participation activities on the subjective well-being of victims of community and structural violence (but not of domestic violence), because, for those who reported higher levels of cultural participation, the probability of better subjective well-being were higher.

**Conclusions:**

Victims potentially coped and adapted to stressful and traumatic situations (i.e., experiences of victimization) via cultural participation activities. However, there are distinctive effects according to different forms of violence, which may be accounted for in formulating public policies related to victims. This has implications for scholars, policymakers, and practitioners in improving the general quality of life of victims and the general population.

## 1. Introduction

The rise of victims in Mexico is an issue of concern. In 2018, Mexico ranked 17th in the homicide rate per one hundred thousand inhabitants, and the 2nd position in absolute scores, at the global level (Muggah and Aguirre, 2018). According to the 2021 Mexican National Survey of Victimization and Perception on Public Security, from 2012 to 2021, the proportion of households that had at least one victim of crime among the family’s members has been 28.4% in average (i.e., a third part of the Mexican households) with the tendency to increase (INEGI, 2021). This propensity has been consistently observed in other sources (see e.g., Corporación Latinobarómetro, n.d.; INEGI, 2019; INEGI, n.d.-a; SESNSP, 2020a).^1^

In Mexico, research of violence and victimization has focused on types of crime, geography of crime, and the characteristics of victims (Cortez, 2015), along with the intersections with gender, poverty, and youth (Maldonado Macedo, 2020; Sanchez and Zhang, 2020; Yates and Leutert, 2020). One potential critical direction in the research of the well-being of Mexicans is the influence of victimization on individuals’ subjective well-being. This research line is relevant because crime and violence in Mexico has been escalating during the last decade (Schedler, 2016; IEP, 2018a,b), along with the number of victims (see e.g., INEGI, 2018; SESNSP, 2020b). It means, to public policy, the need to address the role of victimization on subjective well-being as a central social issue.

Well-being is an important concept in peoples’ life (Organization for Economic Co-operation and Development (OECD), 2011) and a central idea for policymakers in the allocation of public resources (Galloway et al., 2006). However, well-being is still a very challenging concept to define because of its complexity (Galloway et al., 2006; OECD, 2017), which includes a multitude of components (OECD, 2017). To overpass this limitation, several works make use of the subjective well-being dimension –i.e., individuals’ subjective responses to objective conditions (Helliwell and Putnam, 2004), as a discernible component of well-being (OECD, 2013; Blessi et al., 2016; Daykin et al., 2018). Subjective well-being is a helpful concept because of its comparability, validity, and reliability (OECD, 2013). In addition, personal security, violence, and perception of crime have been referred to as potential contributors of subjective well-being (OECD, 2011; Millan and Mancini, 2014; González-König, 2016).

Following literature, in this paper, subjective well-being is composed by two dimensions: cognitive well-being (CWB) and affective well-being (AWB; Angner, 2010; Jovanovic, 2011). The cognitive well-being dimension includes an evaluation of one’s life (or life satisfaction) and happiness (Martinez-Martinez et al., 2018), whereas, the affective balance dimension is observed through positive (e.g., joy and pride) and negative emotions (e.g., pain and worry; Diener and Suh, 1997; Stiglitz et al., 2009; Angner, 2010; Tay et al., 2011).

Concerning victimization –the experience to have been the victim of crime (Dammert and Luneke, 2003; Schedler, 2016), it can be categorized into two broad groups according to (a) the source or type of violence that has elicited the stressful event (i.e., the objective component) or (b) how the event has affected the victim (i.e., the subjective aspect; Echeburúa and Corral, 2007). In this paper, victimization experiences are accounted by the objective component, which includes victimization by domestic violence, community violence, and structural violence. Domestic violence refers to the “intimate partner violence along with family violence” (Barocas et al., 2016), which comprises physical, sexual, and emotional abuse at home (American Academy of Pediatrics, 2020). Victimization by community violence indicates events of “interpersonal violence committed by individuals who are not intimately related to the victim,” such as sexual assault, burglary, muggings, gunshots, and the presence of gangs, and drugs (American Academy of Pediatrics, 2020). Whereas, structural violence is result of unequal economic, political, and social systems, along with ideological or organizational factors that impede the satisfaction of the basic needs of individuals and groups (Jiménez, 2018). For instance, institutionalized adultism, ageism, classism, elitism, ethnocentrism, nationalism, speciesism, racism, and sexism are considered structural violence (Galtung, 1969, cited by Schloerb, 2018).

Victimization has been related, at individual level, to negative influences on personal behavior (Amerio and Roccato, 2007; Averdijk, 2011; Doering and Baier, 2016), life satisfaction (Hanslmaier et al., 2016), general well-being (Di Tella et al., 2008; Hanslmaier, 2013), and physical and mental health (Graham and Chaparro, 2011; Muratori and Zubieta, 2013). At social level, it has been associated to the disruption of family and community life (OECD, 2011; Muratori and Zubieta, 2013), loss of social capital, and detriment of the confidence on government institutions (Di Tella et al., 2008; Graham and Chaparro, 2011). Besides, victimization brings economic costs to individuals, private companies, and governments (INEGI, 2018; IEP, 2019).

Despite these severe consequences, evidence in literature reveals an inaccurate knowledge about victimization experiences and their impact on subjective well-being. There is also an absence of solutions or mechanisms to resolve or attend the effects of victimization on well-being. Therefore, considering the incidence and prevalence of victims in Mexico, it would be of prime concern to delve into the specific effects that victimization brings on victims of crime (Dammert and Luneke, 2003; Diener, 2006). In addition, it would be necessary to understand the strategies and adaptations that persons utilize in response to crime victimization (Green et al., 2010; Moncada, 2020) toward a reintegration to everyday life and, consequently, a restoration of their quality of life and well-being.

Individuals employ several strategies to struggle against the negative effects of violence and crime. Healing through personal empowerment, community healing and empowerment, promoting development, use of culture and spirituality, and democracy building have been argued to counteract the stress and trauma associated with victimization (Van Soest and Prigoff, 1997). Cultural participation^2^ –i.e. participation in cultural and artistic activities– has been described as potential strategy or behavior to cope with the effects of victimization on well-being (Van Soest and Prigoff, 1997; Glover, 1999; Tedeschi, 1999; Pifalo, 2009; Al-Natour, 2013; Marín and Bagan, 2014). Cultural and artistic activities have been regularly assumed to cause positive effects on well-being (Belfiore and Bennett, 2008; Reyes-Martínez et al., 2021), thus, they have been used in public policies and social interventions (Belfiore and Bennett, 2008; Daykin et al., 2018) to alleviate several social problems, such as delinquency and exclusion. To most researchers, cultural participation has a positive impact on quality of life and general well-being (Nenonen et al., 2014; Mundet et al., 2017), subjective well-being (Perkins and Williamon, 2014; Blessi et al., 2016); and health (Daykin et al., 2018). It has also been related to economic benefits (FICAAC, 2005; OECD, 2006; AECID, 2009; UNESCO, 2014) and the building and strengthening of community (Goulding, 2013; Johanson et al., 2014).

Despite all these promising benefits, the evidence in place with regards to how cultural participation works for victims is not well defined yet. Whether cultural participation could play a role between victimization and subjective well-being, or it could be useful as a strategy to minimize the adverse effects of victimization, needs to be investigated. A deeper knowledge regarding this issue will yield in several important implications at scholarship, public policy, and practice level. First, the study of the relationship between cultural participation and the subjective well-being of victims will help to conceptualize into the solutions victims utilize toward a better well-being (Green et al., 2010; Moncada, 2020) and the specific effects that victimization brings on victims (Ley, 2019). To policymakers, results from this research will provide more empirical evidence to include cultural participation in the discussion of the solutions of the effects of victimization, as well as the reinforcement of policies related to public security. Similarly, to practitioners (e.g., social workers), findings from this research will support arguments to incorporate cultural and artistic activities in interventions and programs as tools for social transformation, community building, and democracy promotion.

Bearing that in mind, the purpose of this manuscript is to explore the influence of cultural participation on the subjective well-being of victims, in Mexico. Hopefully, a better comprehension of the problem will allow the reintegration of victims into everyday life and, consequently, a restoration of their subjective well-being.

Therefore, the study advances the next general research question:

(1) What is the influence of cultural participation on the subjective well-being of victims in Mexico?

Besides, the specific research questions are:

(1a) What are the effects of cultural participation on the subjective well-being of the general population?
(1b) What is the influence of self-perceived victimization on the subjective well-being of victims?
(1c) What is the influence of self-perceived victimization on the cultural participation of victims?

## 2. Literature review

### 2.1. Victimization and its impact on subjective well-being and cultural participation

To some scholars, there is a shortage of studies addressing the victimization effects on life satisfaction, affective balance, or happiness (i.e., subjective well-being components; Martínez-Ferrer et al., 2016). This lack of research is particularly acute in developing countries (Cordeiro et al., 2020).

Nevertheless, at the global level, there are some representative analyses that provide us with an outline of the phenomenon. For instance, to some researchers, victimization has a negative impact on all the satisfaction-measures^3^ of subjective well-being (Cordeiro et al., 2020), psychological well-being (Di Tella et al., 2008; Hanslmaier, 2013), and life satisfaction (Graham and Chaparro, 2011; Hanslmaier, 2013; Hanslmaier et al., 2016; Martínez-Ferrer et al., 2016); or a negative correlation with positive emotions and positive correlation with negative emotions (Di Tella et al., 2008). These relationships are modulated by several factors such as adaptation to crime, belonging to a vulnerable group (i.e., according to age or gender), or country’s criminal rate (Graham and Chaparro, 2011); place of residence (Cruz, 1999); type or expression of the experience (e.g., more violent or more direct; Cruz, 1999; Graham and Chaparro, 2011); or income (Di Tella et al., 2008). A less supported position in the literature suggests the absence of an association between both concepts. To a few investigators, due to the lack of statistically significant evidence, victimization does not play a relevant role on individuals’ well-being (Muratori and Zubieta, 2013) or happiness (Ciocchini et al., 2010).

Despite all the evidenced consequences on subjective well-being, several authors support alternative and less-explored theses about the effects of victimization. It means victimization can bring additional outcomes on victims, such as the eliciting of positive emotions (e.g., to develop a new meaning of life), or a potential increment in pro-social behaviors. For instance, to some scholars, crime victimization can increase political participation (Blattman, 2009; Bateson, 2012; Dorff, 2017; Oosterhoff et al., 2018; Page, 2018), civic engagement (Dorff, 2017), social capital (Gilligan et al., 2011), altruistic behavior (Voors et al., 2012), and community leadership (Blattman, 2009). To Sullivan et al. (2010) victimization is also related to other positive social reactions, such as the seeking of services or resources to deal with victimization, as well as the capacity of receiving emotional support (p. 640). In addition, a few researchers suggest that victimization potentially increment the probability of participation in cultural and artistic activities (Jauk, 2013; Reyes-Martínez et al., 2020).

Indeed, in the therapeutic field, scholars have observed how victims rely on the use of arts-related activities to build recovery strategies and release of unacceptable feelings and traumatic events (Glover, 1999; Shuman et al., 2020); identify complex emotions and future risk, develop coping skills (Pifalo, 2009); enhance self-esteem, cope with reality, and reconnect with cultural identity (Al-Natour, 2013); rebuild community and repair safety and trust (Van Soest and Prigoff, 1997).

Besides, cultural participation activities have been used in public policies and social interventions to alleviate several social problems, such as delinquency and exclusion (see e.g., Gobierno del Estado de Guerrero, 2015; SEGOB, 2015), or in the research and understanding of human rights (Adams, 2018).

In spite of the increasing body of literature in the field, these unorthodox theses reveals the need for researching more specific victimization effects and outcomes (Dammert and Luneke, 2003; Ley, 2019) as well as more effective coping strategies (Green et al., 2010).

### 2.2. The role of cultural participation on the subjective well-being of victims

Despite the incidence and prevalence of victimization around the globe, evidence in the extant literature reveals: (a) an inaccurate knowledge about victimization experiences and their impact on subjective well-being, (b) the absence of solutions or mechanisms to resolve or attend the effects of victimization on subjective well-being, and (c) the incomprehension of the role of cultural and artistic activities toward the restoration of the subjective well-being of victims. However, some advances in the field may shed light on the matter (see e.g., Dammert and Luneke, 2003; Green et al., 2010; Ley, 2019; Moncada, 2020; Reyes-Martínez et al., 2020).

For instance, even though victimization is not an absolute determinant of a behavioral change (Averdijk, 2011), some scholars have indicated how conscious or unconscious modifications in routine and behaviors in crime victims can lead toward an improvement or restitution of subjective well-being. For instance, victims rely on the adoption and use of several strategies and actions to deal with the aftermath of traumatic or stressful events (Averdijk, 2011). Some victims change habits or ways of moving, employ self-protective behaviors (e.g., carrying a weapon or any item that can match this use), or follow safety rules, such as avoiding high crime areas or being aware of their surroundings at all times (Frieze et al., 2020).

Cultural participation, as the literature suggests, may be one of the behaviors and strategies victims employ to restitute their subjective well-being. Studies on well-being and cultural participation has emphasized the capacity of cultural and art-related activities to prompt deep and personal emotional reactions (Glover, 1999; Marín and Bagan, 2014) or the development of the communication skills (Mikhaylovsky et al., 2019). Indeed, reparation of victims through artistic processes has been increasingly recognized over the years as alternative restitution methods (Gaitán and Segura, 2017).

To some scholars, in contexts of violence and social crisis, cultural and artistic activities may help victims to overcome depressive symptoms and panic attacks (Bustamante, 2017), process emotions, reconstruct self-stem, promote resilience and empowerment (Moreno, 2016), restore individual and collective identity (Castro, 2016; Moreno, 2016; Bustamante, 2017), reestablish integrity of the individual and the group (Castro, 2016); metabolize conflicts and hopelessness (Petit, 2009), generate positive emotions (Bustamante, 2017), promote creativity and imagination of new realities (Castro, 2016), and foster aesthetic searches (Bustamante, 2017; Reyes-Martínez and Andrade-Guzmán, 2021).

According to Cely-Ávila (2019, p. 33), to victims, it is central to employ embodied and expressive ways of coping and repairing such as artistic resources (e.g., dance, drawing, painting, sculpture, weaving), which allow the reestablishment of emotional ties with one’s own body. For example, in narrative writing, victims relate to the loss and duel in alternative ways, conferring on it new symbolic values through psychological, physical, emotional, relational, and spiritual processes (Bustamante, 2017, p. 98). To Shuman et al. (2020), creative and artistic activities, such as art, play, drama, creative writing, and music are tools to build and cope with the trauma narrative. In interventions oriented to cases of child sexual abuse, arts had been evidenced to reduce trauma-related symptoms, address and promote pro-social behaviors (Shuman et al., 2020). This increase in social behaviors helps to fortify collective identities (Bustamante, 2017). Also in interventions, cultural and artistic activities provides victims strategies of coping to elicit emotions and actions, induce processes of peace, as well as psychological, social, and political empowerment of individuals and communities (Castro, 2016, p. 4).

In other words, cultural participation raises social awareness, and therefore, the consolidation of political, critical, self-critical, and participatory citizens (Castro, 2016, p. 4). It means the effects of participation in arts and culture are not only at the individual level, but also in the building of a more well-being-oriented society.

## 3. Theoretical framework

### 3.1. Theories

This manuscript draws mostly upon the set of coping theories to explore how cultural participation may have an effect on the subjective well-being of victims. In addition, in order to examine the separate relationships between victimization, subjective well-being, and cultural participation, the study looks upon the activity theory, the psychological adaptation approach, and the social contract theory.

In the coping theories, after victimization (i.e., stressful experiences), individuals embrace activities and strategies that are used to restore or recover their well-being and quality of life. Strategies are understood as psychosocial adaptations that individuals implement to manage external and internal demands and where they invest personal resources (Green et al., 2010). Coping strategies help to overpass traumatic experiences from victimization events (Jayawickreme and Blackie, 2014) with the purpose to achieve a better well-being (Green et al., 2010). To several scholars, in contexts of violence and social crisis, cultural and artistic activities may help victims to process emotions, reconstruct identity and self-stem, and promote resilience and empowerment (Moreno, 2016); or in other words, they benefit as mechanisms toward the metabolization of conflicts and hopelessness (Petit, 2009).

The activity theory (Lemon et al., 1972; Nimrod and Adoni, 2006; Rodriguez et al., 2008) is used to account the relationship between cultural participation and subjective well-being. It proposes that individuals who participate in activities are likely to report higher rates of well-being, subjective well-being, or life satisfaction. According to this perspective, physical, intellectual, cultural, and artistic activities are positively associated with subjective well-being.

The connection between victimization and subjective well-being may be addressed by the psychological adaptation theories. Within this framework, the process of adaptation of victims converges, both, on positive and negative effects on victims’ well-being (Hanslmaier et al., 2016; Janssen et al., 2020). It means individuals can adapt themselves easier to some situations than others (Wilson and Gilbert, 2008), which mostly depends on the type and severity of the lived experience (Janssen et al., 2020).

The association between cultural participation and victimization may be informed by the social contract theory. In specific, the approach has been employed to address political behavior and beliefs toward the government in victims of crime (Oosterhoff et al., 2018) and disenfranchised populations (Wray-Lake et al., 2018). However, it could also be accounted to inform pro-social behaviors observed in victims, such as an increment in the participation in cultural and artistic activities (Reyes-Martínez et al., 2020). This last proposition suggests that the social contract theory may be potentially useful to study the relationship between cultural participation and victimization.

### 3.2. Conceptual model

**Figure 1:**
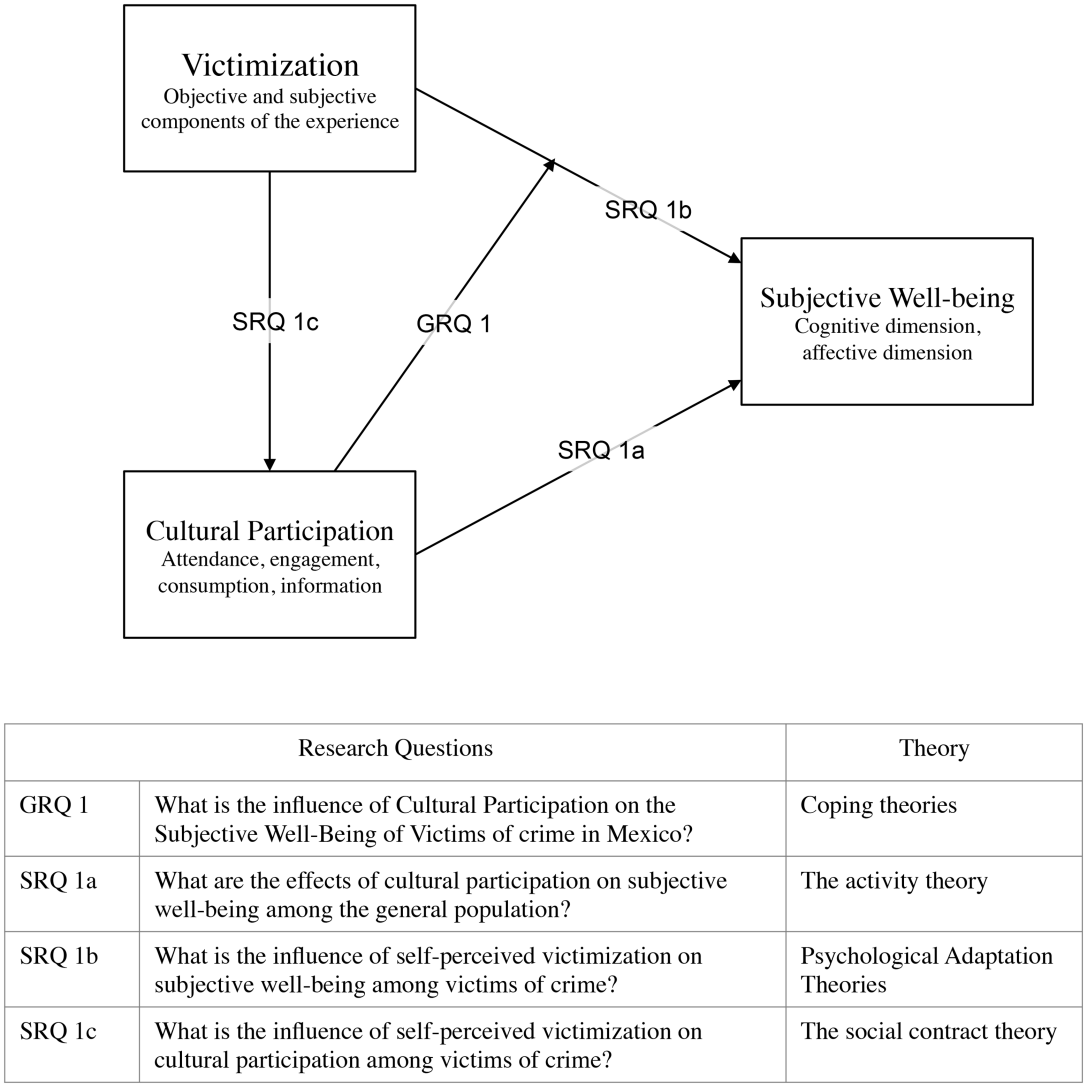
Conceptual model. GRQ1, General Research Question; SRQ1a, Specific Research Question 1a; SRQ1b, Specific Research Question 1b; SRQ1c, Specific Research Question 1c. Proposed theoretical model between victimization, cultural participation, and subjective well-being. Each arrow corresponds with one of the research questions and the theories exposed in the previous subsection.

Consistent with the literature review and the theoretical perspectives, it is possible to propose a theoretical model to answer the research questions in the study. Figure 1 depicts the different relationships between victimization, cultural participation, and subjective well-being and the proposed theoretical approaches to inform them.

For instance, the main research question, namely, the role of cultural participation and its influence on the subjective well-being of victims is represented in the model as a moderator and mediator of the relationship between victimization and subjective well-being.

The other relationships (and research questions) are also illustrated in the model. The association between cultural participation and subjective well-being is depicted as an influencer of subjective well-being; meanwhile, the relationship between victimization and subjective well-being is represented as an influencer (victimization) and outcome (subjective well-being) link. Finally, in the conceptual model, victimization is represented as a potential contributor of cultural participation.

## 4. Hypotheses

Based on the literature review and theoretical framework, we proposed the following four hypotheses:

- H1: Cultural participation will positively influence the subjective well-being of victims, so that at higher levels of cultural participation, the probability of subjective well-being will be higher.
- H1a: Cultural participation enhances the probability of subjective well-being on general population.
- H1b: Self-perceived victimization reduces the probability of subjective well-being among victims.
- H1c: Self-perceived victimization enhances the probability of cultural participation among victims.

## 5. Materials and methods

### 5.1. Study design, dataset, and sampling

This study is a secondary data analysis using the 2012 Self-reported Well-Being Survey (*N* = 10,654; BIARE for *Módulo de Bienestar Autorreportado*, in Spanish). BIARE aims to know how Mexicans experience their quality of life, their current lives, and future perspectives, under their background and environment (INEGI, n.d.-b). It is based on the report of the Commission on the Measurement of Economic Performance and Social Progress (Commission Stiglitz-Sen-Fitoussi; INEGI, n.d.-b). Its design and validation follows recommendations and guidelines by the OECD (see e.g., OECD, 2011) and the European Social Survey.

The 2012 BIARE dataset is representative at the national level for the population between 18 and 70 years old, without territorial disaggregation. The sampling procedure was probabilistic, stratified, two-stage, and by clusters. Each questionnaire was associated with each of the households in the sample of ENGASTO for the first quarter of 2012 (i.e., from January to March 2012). Participants were chosen within members of the selected house using a random method –i.e., the person whose birthday was closer when the survey was conducted in the house. The modality (auto-fill) had a 17% non-response rate; however, 10,654 questionnaires were recovered (INEGI, n.d.-b) and reported in the final dataset. According to the INEGI, all data were weighted regarding the non-response rate.

Most respondents in the survey are female (56.0%). The average age of participants is 39.51 years (standard deviation = 13.85, minimum age = 18 years old, maximum = 70 years old). With regards to educational attainment, 16.6% of the sample indicated no formal schooling, or they completed primary school (19.3%), secondary school (27.4%), high school (18.2%), bachelor (17.1%), and postgraduate education (1.5%). In economic aspects, respondents’ total household income has a mean of 12,090.98 pesos (standard deviation = 16373.80, minimum = 0, maximum = 327586.50).

Regarding missing data, although the dataset does not report any (INEGI, n.d.-b), recoding produced less than 0.05% of lost data. The statistical analysis in this study dealt with missing values using the listwise deletion technique.

Finally, it is important to observe that 2012 BIARE was selected because it is the only dataset in the country that incorporates the variables of interest (see next subsections). More recent versions of the survey do not include indicators related to cultural participation.

### 5.2. Measures

#### 5.2.1. Dependent variables

The main outcome in the research is the self-reported subjective well-being construct. It refers to the responses that individuals provide about objective conditions (Helliwell and Putnam, 2004), and implies people’s evaluations of their life as a whole or in several domains, as well as people’s actual feelings (Stiglitz et al., 2009). Subjective well-being has been usually measured by four indicators associated with the dimensions of cognitive well-being and affective well-being. The cognitive well-being dimension comprises an evaluation of one’s life (or life satisfaction) and happiness. Meanwhile, the affective balance dimension incorporates an assessment of positive (e.g., joy and pride) and negative affects (e.g., pain and worry).

Bearing that in mind, subjective well-being was measured using four interval variables: (1) self-reported life satisfaction (i.e., the cognitive perspective of personal biography), (2) positive emotions (i.e., pleasant affects), (3) negative emotions (i.e., unpleasant affects), and (4) happiness (i.e., how the individual feels in his/her life as a whole, from an emotional perspective; INEGI, n.d.-b). These indicators range from 0 to 10, where 0 is the lower value and 10, the higher.

In the analysis, and considering theoretical and empirical evidence, life satisfaction, happiness, positive emotions, and negative emotions were used in the exploratory and confirmatory factorial analyses to test their role on the cognitive and affective well-being dimensions or factors (see Factorial Analysis Section, p. 12). After that, these dimensions were employed in the GSEM analysis.

#### 5.2.2. Independent variables

This study uses two independent constructs: cultural participation and self-perceived victimization. Cultural participation has been organized according to several practices that incorporate different habits, degrees of involvement, use of time, and expenditure. These criteria have led to several four general types of practices: attendance, engagement, consumption, and information (McCarthy and Jinnett, 2001; NEA, 2009; UNESCO, 2009; ESSnet-CULTURE, 2012). These activities range from more passive to more active practices, as well as economic transactions and the use of mass media (see Introduction section for more details concerning these categories).

In this study, cultural participation was observed through ten dichotomous items organized into three indexes. In the attendance index were included (a) attending concerts, (b) attending movies and theater, and (c) attending museums and galleries. The engagement index incorporated (d) participating in art classes, (e) participating in craft classes, and (f) singing or playing a musical instrument. The consumption index encompassed (g) reading books, (h) reading articles, (i) reading newspapers, and (j) watching educational TV. Selected items are measures of propensity where the respondent indicated whether attended the cultural or artistic activity during the last week or not (0 = no, 1 = yes). According to scholars, measures of propensity do not show qualitative difference between individuals who participate more frequently in cultural and artistic activities and others who participate less frequently (see e.g., Buraimo et al., 2011).

Attendance, engagement, and consumption indexes were built following next steps: 1) items were selected according to availability in the dataset, content validity, unidimensionality, and empirical evidence; 2) each item was weighted equally; and 3) items were aggregated into a single measure (Babbie, 2012). After that, indexes were dichotomized. In the research of cultural participation, the use of dichotomous measurements (and logistic regression models) has been suggested to provide more intuitive results along with better estimates and more reliable assessment of the relationships with other variables.

According to findings in the literature review, each item was included in the exploratory and confirmatory factorial analyses to test their association to the attendance, engagement, and consumption indexes and in the composition of a latent variable. Considering several technical and theoretical criteria (see Factorial Analysis section, p. 12), in the GSEM analysis, indexes were employed to represent the cultural participation construct.

Regarding victimization, it is measured through the self-perceived victimization response (see e.g., OECD, 2011) –i.e., the subjective perception to experiences of crime. Self-perceived victimization has been observed through indicators associated with the objective (i.e., victimization by domestic violence, community violence, school violence, structural violence, cultural violence) and subjective (i.e., direct, indirect, and contextual victimization) components of stressful experiences. In the 2012 BIARE dataset, all available items are based on the categorization by objective components. Thus the self-perceived victimization construct was evaluated using the a) domestic violence, b) community violence, and c) structural violence dimensions. Eighteen dichotomous items integrate these composite variables. Selected indicators specify whether the respondent suffered aggressions and threats at home during last year (0 = no, 1 = yes), experienced aggressions or threats out of home during last year (0 = no, 1 = yes), or suffered mistreatment ever in his or her life (0 = no, 1 = yes) due to structural conditions or not.

Indexes for self-perceived victimization were built following the same steps as those in the cultural participation construct: (1) items were selected according to the availability in the dataset, content validity, unidimensionality, and empirical evidence; (2) each item was weighted equally; and (3) items were aggregated into a single measure (Babbie, 2012). After, resulting indexes were recoded into dichotomous indicators to specify whether or not individuals suffered the reported form of victimization during the last twelve months or ever in his or her life (0 = no, 1 = yes). In criminology, dichotomous measurements have been used to simplify interpretation of results (Farrington and Loeber, 2000). Besides, “the dichotomization of explanatory variables facilitates a ‘risk factor’ approach” useful in the comprehension and prediction of victimization outcomes (Farrington and Loeber, 2000, p. 102).

Regarding the use of these indicators, each individual item was included in the exploratory and confirmatory factorial analyses to test their association with the domestic violence, community violence, and structural violence dimensions. In the GSEM analysis, considering several technical and theoretical issues (see Factorial Analysis section, p. 12), indexes were employed to represent the self-perceived victimization construct.

### 5.3. Statistical analysis

Data were analyzed using univariate analysis, exploratory factorial analysis (EFA), confirmatory factorial analysis (CFA), and Generalized Structural Equation Modeling (GSEM), as well as mediation and moderation testes. All analyses were performed in Stata 15.1.

**Figure 2:**
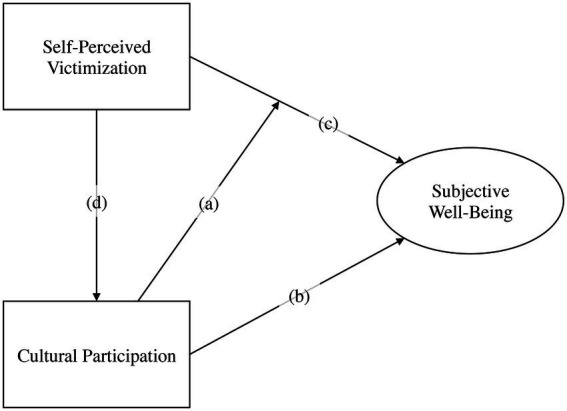
Analytic model. Proposed analytic model among the main constructs: (a) depicts a moderator path or interaction effect [a = b*c]; (b–d) represent direct paths; and (d)*(b) indicates a mediator path or indirect influence of self-perceived victimization on subjective well-being.

Based on the research questions, hypotheses, and conceptual model, the main analysis (i.e., the GSEM approach) explores four relationships: (a) the influence of cultural participation on the relationship between self-perceived victimization and subjective well-being, (b) the relationship between cultural participation and subjective well-being, (c) the influence of self-perceived victimization on subjective well-being, and (d) the association between self-perceived victimization and cultural participation. These relationships are depicted in an analytical model, in Figure 2.

#### 5.3.1. Univariate analysis

Univariate analyses were employed to describe sociodemographic traits of the sample, know the distribution of the variables in the context within the population of reference, and test assumptions of normal distribution (where it applied). Concerning dichotomous variables, relative and absolute frequencies were calculated; while for interval variables, frequencies, mean, standard deviation, variance, skewedness, and kurtosis were also conducted.

#### 5.3.2. Factorial analysis

In general, exploratory factorial analysis (EFA) and Confirmatory factorial analysis (CFA) were employed to model the factors and indexes used in the GSEM analysis.

Regarding the subjective well-being construct, EFA and CFA were performed as a step toward the GSEM test. The use of both techniques was to explore and confirm, respectively, the measurement model suggested by the literature and theory.

In the case of cultural participation and self-perceived victimization variables, both techniques were utilized to define and confirm the structure of the composite indexes. The final decision of using indexes was based on the following criteria: (a) the lack of empirical-based measurement models on the concepts of cultural participation and self-perceived victimization; (b) the need to understand the disaggregated performance of the dimensions of these constructs; and (c), given the exploratory nature of the research, it was preferred the use of a GSEM based on a precision approach, as an alternative to an accuracy approach. In accuracy approaches, those that rely on the use of latent variables, researchers emphasize on the strength of the relations between variables. In comparison, precision approaches, where are preferred the use of observable variables, are used to confirm relationships (see e.g., Ledgerwood and Shrout, 2011).

#### 5.3.3. Generalized structural equation modeling

The Generalized Structural Equation Modeling (GSEM) permits to employ generalized linear models (GLM), such as logistic regression, probit regression, and ordered logistic regression, among others. These features are particularly useful, considering the statistical model in this research combines both dichotomous and interval variables. This type of models can be depicted as in Eq. (1):


(1) $$\eta_{i}=\beta_{0}+\beta_{1}\chi_{1i}+\cdots +\beta_{1}\chi_{pi}$$

and two functions, Eq. (2) a link function that describes how the mean, $Ε\left(Y_{i}\right)=\mu_{i}$ , depends on the linear predictor


(2) $$g\left(\mu_{i}\right)=\eta_{i}$$

and Eq. (3), a variance function that describes how the variance, var.( $Y_{i}$ ) depends on the mean


(3) $$\mathrm{var}\left(Y_{i}\right)=\varphi V\;\left(\mu \right)$$

where the dispersion parameter 𝜑 is a constant (Turner, 2008, p. 15). In the current research, link functions were logit (for binomial variables) and identity (for interval variables).

**Figure 3:**
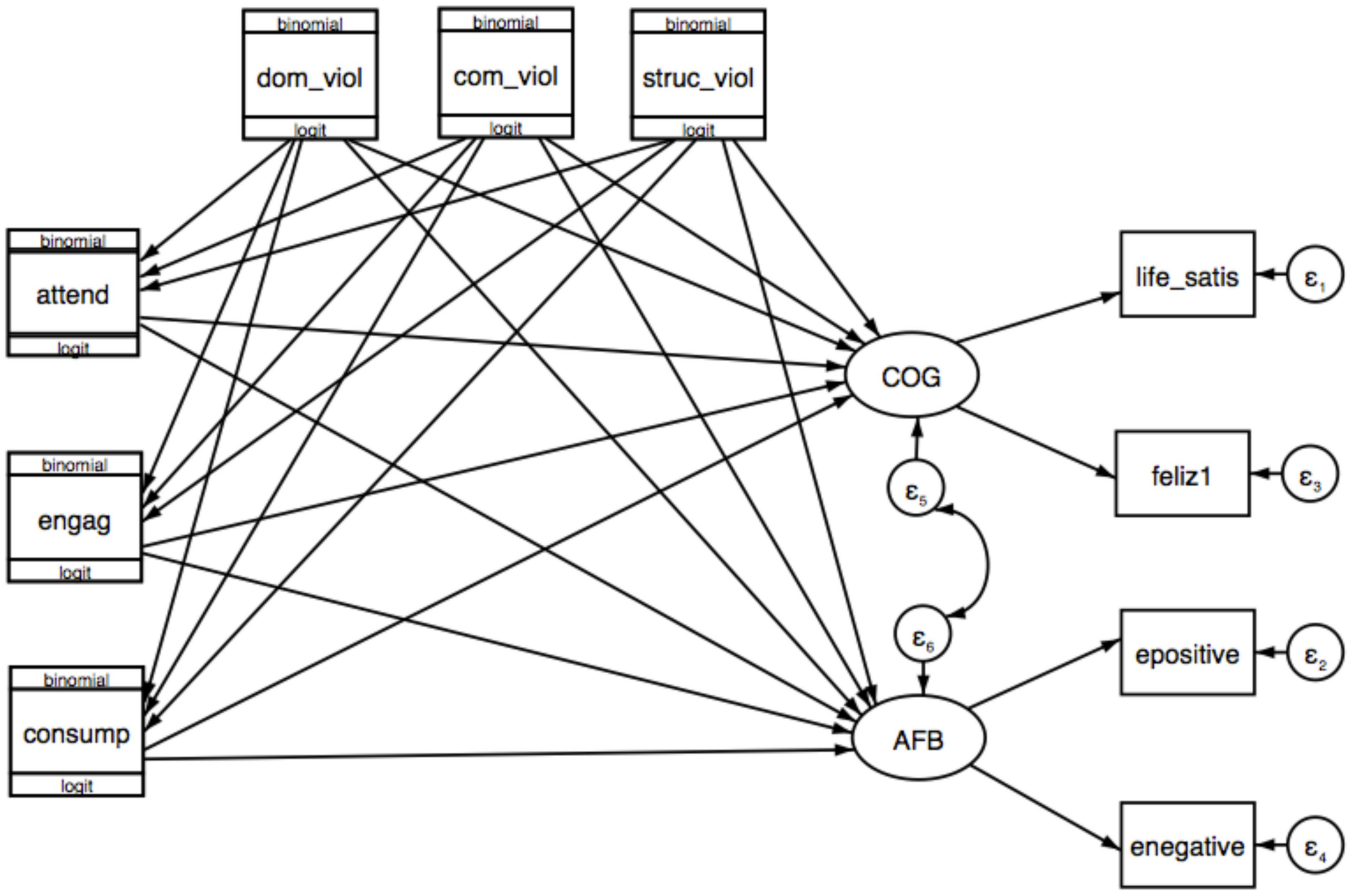
Statistical model. atten, Attendance; engag, Engagement; consump, Consumption; dom_viol, Domestic violence; com_viol, Community violence; struc_viol, Structural violence; life_satis, Life satisfaction; feliz, Happiness; epositive, Positive emotions; enegative, Negative emotions; COG, Cognitive well-being; AFB, Affective balance. This diagram does not include moderation (or interaction terms) paths.

Besides, the measurement model is composed by life satisfaction, happiness, positive emotion, and negative emotion variables that are associated with the cognitive well-being and affective balance latent variables (both, as dimensions of subjective well-being). These latent constructs were also the main outcomes in the structural model, which in turn includes the indexes of cultural participation and self-perceived victimization. Figure 3 depicts the final statistical GSEM model.

Lastly, the GSEM analysis relies on nonadaptive Gauss–Hermite quadrature technique, with 7 integration (quadrature) points. In addition, considering GSEM does not allow for some post-estimation tests (in comparison to SEM), calculations were not performed.

#### 5.3.4. Moderation and mediation effects

Interactions tests are performed to evaluate the moderation effect of cultural participation construct on the relationship between self-perceived victimization and subjective well-being. In the research, Hypothesis 1 describes a potential moderation effect of cultural participation, where self-perceived victimization (*X*) effects subjective well-being (*Y*), but victimization (*X*) changes in relation to variations on cultural participation (*Z*). This association can be represented as indicated in Eq. (4):


(4) $$Y=b_{0}+b_{1}X+b_{2}Z+b_{3}XZ+e$$

where

*b* = Changes in slope by the variable

*Y* = Dependent variable

*X* = Independent variable

*Z* = Moderator variable

*XZ* = Product term between *X* and *Z*

e = error

In this equation *XZ* represents the interaction effect between self-perceived victimization and cultural participation. The coefficient *b_3_* indicates the change in the slope of the regression of self-perceived victimization –> subjective well-being, when cultural participation changes by one unit (Lyytinen and Gaskin, n.d.).

Alternatively, Hypothesis 1 was interpreted as a mediation relationship, where self-perceived victimization may have an indirect effect on subjective well-being *via* cultural participation. In this situation, cultural participation operates as an intervening variable. To test whether a mediation effect exists or not, a four-step approach was employed (see Table A1, in Appendix A; Baron and Kenny, 1986). In addition, to calculate indirect effects of the predictor (i.e., self-perceived victimization) a Sobel’s test was conducted. After mediation was determined, next step implied calculating the indirect effects of mediation using the Sobel’s test (Eq. 5), which can be represented as follows:


(5) $$b_{\mathrm{indirect}}=\left(b_{2}\right)\left(b\right)\;$$

where

*b*_indirect_ = Indirect effect of the predictor (i.e., self-perceived victimization).

*b_2_ =* Partial regression coefficient for cultural participation (M) predicting subjective well-being (Y).

*b =* Simple regression coefficient for self-perceived victimization (X) predicting cultural participation (M) (Newsom, n.d.).

Finally, the total effects of self-perceived victimization indicators were also calculated as follows, in Eq. (6):


(6) $$b_{\mathrm{total}}=c+ab$$

where

*b*_total_ = Total effect of the predictor (i.e., each self-perceived victimization index).

*a* = Regression coefficient for self-perceived victimization (*X*) predicting cultural participation (*M*).

*b* = Regression coefficient for cultural participation (M) predicting subjective well-being (Y).

*ab* = Product of a and b (the indirect effect).

*c* = Regression coefficient for self-perceived victimization (*X*) predicting subjective well-being (*Y*) (or the direct effect) (Baron and Kenny, 1986).

## 6. Findings

### 6.1. Univariate analysis results

The descriptive statistics of subjective well-being, self-perceived victimization, and cultural participation variables are summarized in Appendix A, Tables A2, A3, and A4, respectively.

### 6.2. Factorial analysis results

Table 1 summarizes CFA for the subjective well-being items^4^. All standardized factor loadings were statistically significant (*p* < 0.001). As expected, life satisfaction, happiness, and positive emotions reported positive scores, whereas negative emotions reported a negative one. Table 1 also displays results for the measurement error variances. In the proposed model, standardized measurement error variance ranged from 0.18 to 0.88. Regarding covariance, latent variables show significant and positive standardized values (0.76). These scores indicate that both factors are highly and positively correlated, which is coherent with literature and theoretical foundations of the subjective well-being construct.

**Table 1:** CFA estimates, subjective well-being variables.

Measurement	Coeff.^a^	Std. Err.	*z*	*|p| > z*
Positive emotions				
Affective balance	0.9005***	0.0157	57.22	0.000
Constant	3.6887***	0.0271	136.30	0.000
Negative emotions				
Affective balance	−0.3447***	0.0104	−33.06	0.000
Constant	1.2574***	0.0130	96.99	0.000
Happiness				
Cognitive well-being	0.7050***	0.0074	95.42	0.000
Constant	4.7887***	0.0342	140.00	0.000
Life satisfaction				
Cognitive well-being	0.7655***	0.0072	105.68	0.000
Constant	4.2605***	0.0308	138.54	0.000
var(e.positive emotions)	0.1891***	0.0283	–	0.000
var(e.negative emotions)	0.8812***	0.0072	–	0.000
var(e.happiness)	0.5029***	0.0104	–	0.000
var(e.life satisfaction)	0.4141***	0.0111	–	0.000
var(affective balance)	1	–	–	–
var(cognitive well-being)	1	–	–	–
cov(affective balance, cognitive well-being)	0.7616***	0.0148	51.33	0.000
*N*	10,654			
ll	−86872.01			
*p*	0.000			
chi2 (1)	23.48			
aic	173770.02			
bic	173864.58			

a, Standardized coefficient. ***p < 0.001. –, Not available. Author’s elaboration.

Table 2 displays several model-fit criteria. For instance, chi-square tests were statistically significant (*p* < 0.001). The model also presented adequate levels for RMSEA and SRMR (see e.g., Schumacker and Lomax, 2016). Table 2 also shows R-square values. In the proposed model, the r-square values ranged from 11 to 81%, and the overall variance explained by the model is 91%.

**Table 2:** CFA post-estimates, subjective well-being variables.

Criteria	Values
Fit statistics	
Chi-Square	9102.04
p > chi2	0.000
Degrees of freedom	6
RMSEA	0.05
Akaike information criterion (AIC)	173770.02
Bayesian information criterion (BIC)	173864.58
Comparative Fit Index (CFI)	0.99
Tucker-Lewis index (TLI)	0.98
Standardized RMR (SRMR)	0.01
Coefficient of Determination (CD)	0.91
R2	
Life satisfaction	0.58
Happiness	0.49
Positive emotions	0.81
Negative emotions	0.11
Overall	0.91

Author’s elaboration.

Finally, Table 3 depicts the alpha reliability scores for each dimension of subjective well-being, as well as the total score for the whole set of items (0.6774).

**Table 3:** Alpha reliability, subjective well-being variables and dimensions.

Dimension	Variables	Scale reliability coefficient	Average interitem covariance
Cognitive well-being	Life satisfaction	0.6997	1.7851
	Happiness		
Affective balance	Positive emotions	0.4662	1.7128
	Negative emotions		
Total	All variables	0.6774	1.5410

Author’s elaboration.

In the case of self-perceived victimization and cultural participation, EFA and CFA were also run. However, considering several criteria, indexes were used instead of latent variables (see Factorial Analysis subsection, p. 15), so, results for these tests are omitted here.

### 6.3. GSEM results

Table 4 presents the GSEM results for the statistical model. Concerning the measurement model, regression analysis showed statistically significant associations with the cognitive well-being and affective balance latent variables. Indeed, life satisfaction and happiness showed a positive relationship with cognitive well-being (*p* < 0.001), whereas positive emotions and negative emotions indicated a positive and negative relationship, respectively, with affective balance (*p* < 0.001). Furthermore, the covariance between cognitive well-being and affective balance is significant and positive (*p* < 0.001) which confirms an association between both latent variables.

**Table 4:** GSEM analysis results.

Variables	Coef. ^a^	OR	Std. Err.	*z*	*p > |z|*	[95% Conf. Interval]	
Cognitive well-being							
Domestic violence	−1.2467***	0.2875***	0.1684	−7.40	0.000	−1.5767	−0.9166
Community violence	−0.9381***	0.3914***	0.1053	−8.91	0.000	−1.1444	−0.7317
Structural violence	−0.9351***	0.3926***	0.0901	−10.38	0.000	−1.1117	−0.7584
Attendance	0.1264**	1.1347**	0.0411	3.07	0.002	0.0458	0.2070
Engagement	0.1248**	1.1329**	0.0468	2.67	0.008	0.0331	0.2165
Consumption	0.2794***	1.3223***	0.0393	7.11	0.000	0.2024	0.3564
c.attendance # c.domestic_viol	−0.2422	0.7849	0.1716	−1.41	0.158	−0.5786	0.0942
c.engagement # c.domestic_viol	0.3844*	1.4688*	0.1777	2.16	0.031	0.0361	0.7328
c.consumption # c.domestic_viol	0.0882	1.0922	0.1807	0.49	0.625	−0.2660	0.4424
c.attendance # c.community_viol	−0.0831	0.9202	0.1003	−0.83	0.407	−0.2796	0.1134
c.engagement # c.community_viol	0.1487	1.1603	0.1118	1.33	0.184	−0.0705	0.3678
c.consumption # c.community_viol	0.6557***	1.9265***	0.1185	5.53	0.000	0.4234	0.8880
c.attendance # c.structural_viol	0.6775***	1.9689***	0.0956	7.09	0.000	0.4901	0.8648
c.engagement # c.structural_viol	0.0701	1.0726	0.1042	0.67	0.501	−0.1342	0.2743
c.consumption # c.structural_viol	−0.0826	0.9207	0.1034	−0.80	0.424	−0.2852	0.1200
Affective balance							
Domestic violence	−0.9908***	0.3713***	0.2349	−4.22	0.000	−1.4511	−0.5305
Community violence	−1.0999***	0.3329***	0.1159	−9.49	0.000	−1.3271	−0.8727
Structural violence	−1.3438***	0.2609***	0.0799	−16.82	0.000	−1.5004	−1.1872
Attendance	0.1508***	1.1628***	0.0325	4.64	0.000	0.0871	0.2146
Engagement	0.0576	1.0592	0.0368	1.56	0.118	−0.0146	0.1297
Consumption	0.1328***	1.1420***	0.0371	3.58	0.000	0.0602	0.2055
c.attendance # c.domestic_viol	−0.4869**	0.6145**	0.1543	−3.15	0.002	−0.7894	−0.1844
c.engagement # c.domestic_viol	0.3971*	1.4875*	0.1708	2.33	0.020	0.0624	0.7318
c.consumption # c.domestic_viol	−0.3007	0.7403	0.2222	−1.35	0.176	−0.7361	0.1348
c.attendance # c.community_viol	0.0228	1.0231	0.0972	0.23	0.814	−0.1677	0.2133
c.engagement # c.community_viol	0.1744	1.1905	0.0986	1.77	0.077	−0.0190	0.3677
c.consumption # c.community_viol	0.8148***	2.2587***	0.1289	6.32	0.000	0.5621	1.0674
c.attendance # c.structural_viol	1.2247***	3.4030***	0.0921	13.30	0.000	1.0442	1.4051
c.engagement # c.structural_viol	−0.0809	0.9223	0.0873	−0.93	0.354	−0.2519	0.0901
c.consumption # c.structural_viol	−0.0274	0.973	0.0910	−0.30	0.764	−0.2058	0.1511
Attendance							
Domestic violence	−0.1941	0.8235	0.1129	−1.72	0.085	−0.4154	0.0271
Community violence	0.3831***	1.4668***	0.0657	5.83	0.000	0.2543	0.5118
Structural violence	0.4109***	1.5082***	0.0627	6.55	0.000	0.2880	0.5339
Constant	−1.3996***	–	0.0277	−50.55	0.000	−1.4539	−1.3453
Engagement							
Domestic violence	0.1492	1.1609	0.1179	1.26	0.206	−0.0820	0.3803
Community violence	0.2853***	1.3302***	0.0749	3.81	0.000	0.1385	0.4322
Structural violence	0.3907***	1.4780***	0.0707	5.53	0.000	0.2521	0.5292
Constant	−1.8356***	–	0.0319	−57.47	0.000	−1.8982	−1.7730
Consumption							
Domestic violence	−0.1659	0.8471	0.1080	−1.54	0.124	−0.3775	0.0457
Community violence	0.3169***	1.3729***	0.0753	4.21	0.000	0.1693	0.4645
Structural violence	0.1683*	1.1833*	0.0694	2.42	0.015	0.0322	0.3044
Constant	1.1831***	–	0.0263	45.06	0.000	1.1316	1.2345
Life satisfaction							
Cognitive well-being	1	–	–	–	–	–	–
Constant	8.0755***	–	0.0353	228.82	0.000	8.0064	8.1447
Happiness							
Cognitive well-being	0.8712***	2.3898***	0.015	58.18	0.000	0.8418	0.9005
Constant	8.3978***	–	0.0313	268.48	0.000	8.3365	8.4591
Positive emotions							
Affective balance	1	–	–	–	–	–	–
Constant	8.0878***	.	0.0357	226.74	0.000	8.0179	8.1577
Negative emotions							
Affective Balance	−0.4051***	0.6669***	0.0121	−33.46	0.000	−0.4288	−0.3814
Constant	3.1619***	–	0.0280	112.86	0.000	3.1069	3.2168
var(e.Cognitive well-being)	1.7505***	5.7576***	0.0451	38.79	0.000	1.6621	1.8390
var(e.Affective Balance)	4.0359***	56.5950***	0.0380	106.32	0.000	3.9615	4.1103
var(e.life satisfaction)	1.6174***	5.0401***	0.0343	47.13	0.000	1.5502	1.6847
var(e.happiness)	1.5768***	4.8392***	0.0301	52.40	0.000	1.5178	1.6357
var(e.positive emotions)	0.2902***	1.3367***	0.0097	29.96	0.000	0.2712	0.3092
var(e.negative emotions)	6.0742***	434.4947***	0.0841	72.22	0.000	5.9093	6.2390
Cov(e.CWB, e.AWB)	1.9627***	7.1185***	0.0311	63.11	0.000	1.9017	2.0237
*N*	10,573						
ll	−101375.60						
df	55						
aic	202861.30						
bic	203260.90						

a, Unstandardized coefficient. *p < 0.05, **p < 0.01, ***p < 0.001. –, Not available.

In the structural model, several relations were estimated. Regarding the cognitive well-being construct, all self-perceived victimization variables (domestic violence, community violence, and structural violence) showed statistically significant and negative associations with it (*p* < 0.001). In comparison, the cultural participation variables (attendance, engagement, and consumption) indicated a significant but positive relationship with cognitive well-being, at different significance levels (*p* < 0.001 and *p* < 0.01). Regarding the affective balance construct, all self-perceived victimization variables specified significant and negative associations with the latent variable (*p* < 0.001). In the case of cultural participation variables, only attendance and consumption showed significant and positive relationships with affective balance (*p* < 0.001). Engagement did not report a significant association with affective balance.

Table 4 also reports the link between self-perceived victimization and cultural participation variables. Domestic violence estimates indicated non-significant relationships with the cultural participation variables. Contrary, community violence showed statistically significant and positive associations with attendance, engagement, and consumption (*p* < 0.001). Similarly, structural violence presented significant and positive relations with cultural attendance and engagement (*p* < 0.001), and cultural consumption (*p* < 0.05).

**Figure 4:**
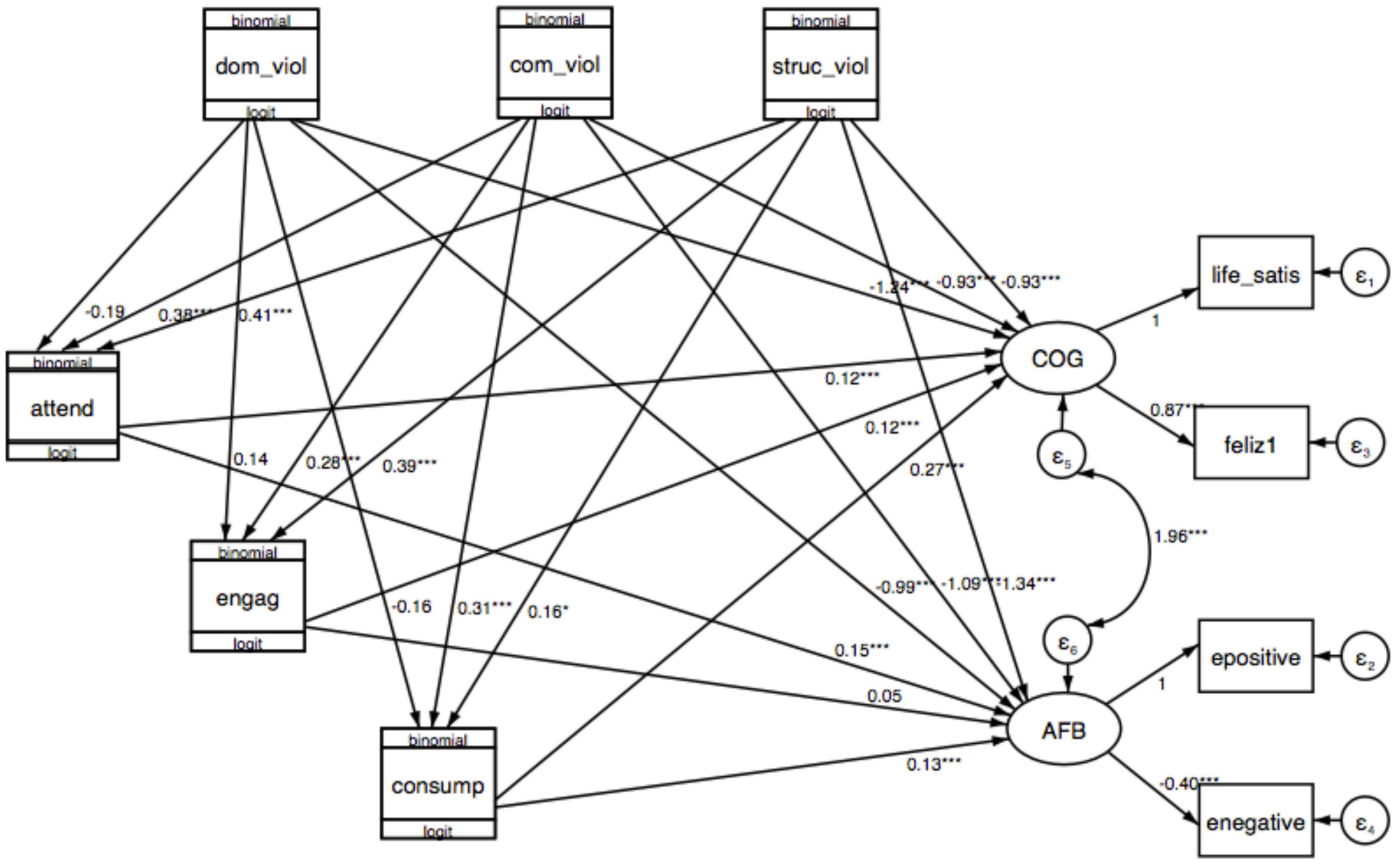
Final statistical model, unstandardized coefficients^a^. a, Interaction terms are not included; atten, Attendance; engag, Engagement; consump, Consumption; dom_viol, Domestic violence; com_viol, Community violence; struc_viol, Structural violence; life_satis, Life satisfaction; feliz, Happiness; epositive, Positive emotions; enegative, Negative emotions; COG, Cognitive well-being; AFB, Affective balance. *p < 0.05, **p < 0.01, ***p < 0.001.

Figure 4 presents the final statistical model with the estimates for the measurement and structural model.

### 6.4. Moderation and mediation results

Table 4 also depicts results of the moderation effects of the cultural participation variables on the relationships between self-perceived victimization indicators and subjective well-being dimensions (i.e., cognitive well-being and affective balance).

Concerning effects on cognitive well-being, only the following interaction terms presented statistically significant and positive estimates: (a) engagement influencing on domestic violence path (*p* < 0.05); (b) consumption on the community violence path (*p* < 0.001); and (c) attendance on the structural violence path (*p* < 0.001). Similarly, in the case of affective balance, from nine hypothesized associations, only four of them were significant: (a) attendance moderating domestic violence path (*p* < 0.01); (b) engagement on domestic violence (*p* < 0.05); consumption on community violence (p < 0.001); and attendance on structural violence (*p* < 0.001). Of these, the first reported a negative direction, while the other three, a positive one.

Regarding mediation effects, we performed the four-step approach suggested by Baron and Kenny (1986) (see Table A1, in Appendix A), which was followed by the Sobel’s test (see Table 5). As observed, all indirect effects of domestic violence *via* the cultural participation indicators were not significant. Contrary, most coefficients from the influence of community violence and structural violence were statistically significant and positive, at different significance levels (*p* < 0.05, *p* < 0.01, *p* < 0.001). One exception came from the influence of self-perceived victimization variables on affective balance *via* engagement that was also not significant.

**Table 5:** Mediation test results, indirect effects of self-perceived victimization variables on subjective well-being dimensions, *via* cultural participation variables.

Variables	Coef. ^a^	OR	Std. Err.	*z*	*p > |z|*	[95% Conf. Interval]	
Domestic violence							
*via* Attendance to Cognitive well-being	−0.0245	0.9757	0.0164	−1.50	0.133	−0.0566	0.0075
*via* Engagement to Cognitive well-being	0.0186	1.0187	0.0163	1.14	0.253	−0.0133	0.0505
*via* Consumption to Cognitive well-being	−0.0464	0.9547	0.0309	−1.50	0.133	−0.1068	0.0141
*via* Attendance to Affective balance	−0.0293	0.9711	0.0182	−1.61	0.107	−0.0649	0.0063
*via* Engagement to Affective balance	0.0086	1.0086	0.0087	0.98	0.325	−0.0085	0.0257
*via* Consumption to Affective balance	−0.0220	0.9782	0.0156	−1.41	0.158	−0.0526	0.0085
Total indirect effect on Cog. well-being	−0.0523	0.9490	0.0387	−1.35	0.177	−0.1281	0.0236
Total indirect effect on Affective balance	−0.0427	0.9581	0.0256	−1.67	0.095	−0.0929	0.0074
Total indirect effect of Domestic violence	−0.0950	0.9093	0.0617	−1.54	0.123	−0.2159	0.0258
Community violence							
*via* Attendance to Cognitive well-being	0.0484**	1.0496	0.0178	2.72	0.007	0.0135	0.0833
*via* Engagement to Cognitive well-being	0.0356*	1.0362	0.0163	2.18	0.029	0.0037	0.0676
*via* Consumption to Cognitive well-being	0.0885***	1.0925	0.0244	3.62	0.000	0.0406	0.1365
*via* Attendance to Affective balance	0.0578***	1.0594	0.0159	3.63	0.000	0.0266	0.0890
*via* Engagement to Affective balance	0.0164	1.0165	0.0113	1.45	0.148	−0.0058	0.0387
*via* Consumption to Affective balance	0.0421**	1.0429	0.0154	2.73	0.006	0.0119	0.0723
Total indirect effect on Cog. well-being	0.1726***	1.1883	0.0317	5.44	0.000	0.1104	0.2347
Total indirect effect on Affective balance	0.1163***	1.1233	0.0226	5.15	0.000	0.0720	0.1606
Total indirect effect of Comm. violence	0.2889***	1.3349	0.0494	5.85	0.000	0.1921	0.3856
Structural violence							
*via* Attendance to Cognitive well-being	0.0519**	1.0533	0.0187	2.78	0.005	0.0154	0.0885
*via* Engagement to Cognitive well-being	0.0488*	1.0499	0.0203	2.40	0.016	0.0090	0.0885
*via* Consumption to Cognitive well-being	0.0470*	1.0481	0.0205	2.29	0.022	0.0068	0.0872
*via* Attendance to Affective balance	0.0620***	1.0639	0.0164	3.78	0.000	0.0299	0.0941
*via* Engagement to Affective balance	0.0225	1.0227	0.0149	1.51	0.132	−0.0068	0.0518
*via* Consumption to Affective balance	0.0224*	1.0226	0.0111	2.01	0.045	0.0005	0.0442
Total indirect effect on Cog. well-being	0.1477***	1.1591	0.0318	4.65	0.000	0.0854	0.2100
Total indirect effect on Affective balance	0.1068***	1.1127	0.0227	4.71	0.000	0.0624	0.1513
Total indirect effect of Structural violence	0.2545***	1.2898	0.0488	5.21	0.000	0.1588	0.3502

a, Unstandardized coefficient. Author’s elaboration. *p < 0.05, **p < 0.01, ***p < 0.001.

In addition, Table 5 shows the total indirect effects of self-perceived victimization variables on the subjective well-being dimensions. As observed, except for domestic violence, results suggest that community and structural violence had an indirect effect on cognitive well-being and affective balance, *via* the cultural participation indexes. All these total indirect effects were statistically significant and positive (*p* < 0.001).

Along with indirect effects, it was also relevant to calculate the total effect of the self-perceived victimization variables on the subjective well-being dimensions. Table 6 shows the total effect (i.e., the sum of direct and indirect effects) for domestic violence, community violence, and structural violence.

**Table 6:** Mediation test results, total effects of self-perceived victimization variables on subjective well-being dimensions.

Variables	Direct effect^a^	Indirect effect^a^	Total effect (Indirect + Direct effect)				
			Coef.^a^	OR	Std. Err.	*z*	*p > |z|*
Domestic violence							
Total on Cognitive well-being	−1.2467***	−0.0523	−1.2990***	0.2728	0.1721	−7.55	0.000
Total on Affective balance	−0.9908***	−0.0427	−1.0335***	0.3557	0.2357	−4.38	0.000
Total of Domestic violence	−2.2375***	−0.0950	−2.3325***	0.0970	0.3724	−6.26	0.000
Community violence							
Total on Cognitive well-being	−0.9381***	0.1726***	−0.7655***	0.4651	0.1125	−6.80	0.000
Total on Affective balance	−1.0999***	0.1163***	−0.9836***	0.3740	0.0879	−13.97	0.000
Total of Community violence	−2.0380***	0.2889***	−1.9930***	0.1362	0.1472	−13.54	0.000
Structural violence							
Total on Cognitive well-being	−0.9351***	0.1477***	−0.7873***	0.4550	0.0976	−8.07	0.000
Total on Affective balance	−1.3438***	0.1068***	−1.2370***	0.2902	0.0864	−14.32	0.000
Total of Structural violence	−2.2789***	0.2545***	−2.0243***	0.1320	0.1546	−13.10	0.000

a, Unstandardized coefficient. Author’s elaboration. *p < 0.05, **p < 0.01, ***p < 0.001. Not available.

As noted in Table 6, all coefficients were statistically significant and negative (*p* < 0.001). Besides, it is possible to observe that domestic violence, despite it had a significant direct influence on cognitive well-being and affective balance, it did not report significant indirect influence.

In community violence and structural violence, direct effects on cognitive well-being and affective balance were greater than total effects, suggesting a positive influence of the cultural participation variables as mediators in the relationship. In other words, the presence of cultural participation lessened the negative effects of the victimization experiences on subjective well-being.

## 7. Discussions, conclusions, and limitations

### 7.1. Discussions

#### 7.1.1. The role of cultural participation

As previous research suggests, participation in cultural and artistic activities may lessen the negative effects of experiences of victimization on the subjective well-being of individuals, in comparison to those who do not participate (see e.g., Shuman et al., 2020). Statistical findings along with the proposed theoretical framework support the idea that victims tend to rely on several strategies to manage stressful and traumatic events (see e.g., Averdijk, 2011). These strategies point to culture and arts-related activities as components of cognitive and emotional mechanisms toward the restoration of the personal subjective well-being.

Experiences of victimization elicit a vast array of emotions that eventually lead to an increase in pro-social behaviors and other forms of individual and collective participation, such as those based on cultural and artistic activities (see e.g., Bateson, 2012; Dorff, 2017; Oosterhoff et al., 2018; Nussio, 2019). However, the potential influence of self-perceived victimization on cultural participation (see e.g., Reyes-Martínez et al., 2020) and the influence of some types of cultural participation on the effects of victimization have been scarcely supported (see e.g., Bustamante, 2017; Gaitán and Segura, 2017). So, it could open to new directions and research lines in the understanding of victimization episodes, as well as in the solutions of the negative effects of these experiences.

Results here suggest that all dimensions of cultural participation (i.e., attendance, engagement, and consumption) may moderate on the effects of specific types of violence toward distinctive subjective well-being dimensions. Although most statistical associations reported a positive direction, a negative effect –i.e., the interaction between attendance and domestic violence toward affective balance– also emerged in these findings. Both positions are coherent with previous research. According to some scholars, cultural participation may bring mixed effects on subjective well-being when differentiated dimensions are analyzed (see e.g., Daykin et al., 2008, 2018). It means, cultural and artistic activities can positively and negatively contribute on general well-being (Hampshire and Matthijsse, 2010). Only to a few researchers, some cultural activities may lead to negative outcomes (e.g., sadness or psychological stress) on subjective well-being (Dockery, 2011; Biddle and Crawford, 2017). Besides, findings insinuate cultural attendance activities (i.e., a more passive form of participation) may worsen the impact of domestic violence on the affective balance dimension of some individuals. Conceivably, this may occur because these victims confront with traumatic or disturbing narratives, employ arts as mediums to canalize painful experiences (Dockery, 2011; Biddle and Crawford, 2017), or use maladaptive coping processes (Zhang and Noels, 2013).

In addition to exerting an influence as a moderator of victimization, some cultural participation activities may play the role of mediators in the relationship between some expressions of self-perceived victimization and cognitive well-being and affective balance.

Unlike current research in the field, results indicate distinctive outcomes in the role of the cultural participation as mediator of the relationship between self-perceived victimization and subjective well-being dimensions. In literature, victimization has been evidenced to have a negative influence on subjective well-being (Cordeiro et al., 2020), life satisfaction (Hanslmaier et al., 2016; Martínez-Ferrer et al., 2016), and positive emotions (Di Tella et al., 2008). However, it has also been identified that effects from victimization could be shaped by the type of experience (Cruz, 1999; Graham and Chaparro, 2011) and belonging to vulnerable groups (e.g., according to age, gender or race; see e.g., Echeburúa and Corral, 2007; Frieze et al., 2020). It means it is possible that victimization events could lead to unique outcomes depending on other moderating factors, such as the participation in cultural and artistic activities.

For instance, despite the extensive research concerning intimate violence, the underlying mechanisms that motivate domestic violence and behaviors of victims has been scarcely investigated, and thus, they are not fully understood (Shackelford and Hansen (eds.) 2014). To some scholars, domestic violence victims rely more on formal or informal support networks or self-help groups to get support (Miracco et al., 2010). In the same way as with the moderation effect, in mediation, victims of domestic violence may employ maladaptive strategies such as avoidance, consent, and isolation (Molina and Moreno, 2015) that may lead to the null use of alternative coping tools, such as the artistic and cultural activities.

Besides, results from the total effect of the self-perceived victimization variables on the subjective well-being dimensions support the idea that cultural attendance, engagement, and consumption may lessen the deleterious effects of community and structural violence on cognitive well-being and affective balance, which is not the situation of domestic violence. Thus, it is not possible to state that the benefits of cultural participation activities apply in all situations or experiences of victimization.

Concerning the theoretical framework, as preceding researches suggest, propositions and concepts in the set of coping theories are coherent with the proposed theoretical model. Coping theories can be helpful informing the positive influence of cultural participation on the subjective well-being of those individuals who has experienced community and structural violence. In conceptual terms, cultural participation can be accounted as a coping strategy that mediates and moderates the relationship between self-perceived victimization and subjective well-being.

Previous research suggests why there are dissimilarities between several forms of victimization and cultural activities. In the coping theories, individuals employ solutions according to how they appraise events and the availability of personal and social resources (Zimmer-Gembeck and Skinner, 2016). This argument could explain the reasons some individuals undertook different strategies and experienced different effects (Frieze et al., 2020).

#### 7.1.2. Other relationships

As previous research suggests, cultural participation is positively associated with subjective well-being (see e.g., Daykin, 2020). In specific, in the case of Mexicans, attendance, engagement, and consumption can be considered potential contributors of cognitive well-being; whereas, only attendance and consumption can be related to affective balance. These results are coherent with the most central position in the literature. To most scholars, cultural participation has been evidenced to produce a positive impact on subjective well-being (Toepoel, 2011; Blessi et al., 2016; Mundet et al., 2017; Daykin et al., 2018).

A suitable explanation for these outcomes lies in the activity theory. According to it, individuals who participate in activities are likely to report higher rates of psychological well-being, subjective well-being, or life satisfaction (Joung and Miller, 2007). It occurs because, faced with new situations and contexts, individuals adjust and change its roles and behaviors. These modified routines help to preserve an integral self-concept, leading to well-being and life satisfaction (Joung and Miller, 2007).

Regarding the association between self-perceived victimization and subjective well-being, similar to previous studies, analysis showed a significant and negative relationship between domestic violence, community violence, and structural violence, and cognitive well-being and affective balance. It suggests that self-perceived victimization diminishes the probability of better subjective well-being.

To scholars, victimization has been evidenced to bring negative impacts on several satisfaction domains, life-satisfaction, subjective well-being, and positive emotions (Di Tella et al., 2008; Graham and Chaparro, 2011; Hanslmaier et al., 2016; Cordeiro et al., 2020), as well as positive effects on negative emotions (Di Tella et al., 2008). The psychological adaptation theories are a helpful set of approaches to inform this relationship. Under this perspective, adaptation is the capacity of adjustment and acceptance as well as the process of recuperation after a setback (Heyink, 2016).

According to scholars, individuals can adapt (or not) in an easy way to some circumstances than to others (Wilson and Gilbert, 2008). But this mostly depends on the type of victimization (see e.g., Cruz, 1999; Graham and Chaparro, 2011; Janssen et al., 2020). Thus, a poor or incomplete adaptive process from the victimization experiences may occur because of several moderators not observed, such as the social context, time from the experience, previous level of well-being, individual’s expectations, mental health situation, or personality (Heyink, 2016).

In regards to the relationship between self-perceived victimization and cultural participation, in a similar way as the few preceding research, results reveal a positive relationship between community violence and structural violence and cultural attendance, engagement, and consumption. Contrary, domestic violence did not show a statistical association with cultural participation. In other words, those who have reported experiences of victimization had a higher probability of participating in cultural and artistic activities.

To some researchers, victimization can also bring to individuals a potential increment in pro-social behaviors, such as political participation, civic engagement (Blattman, 2009; Gilligan et al., 2011; Bateson, 2012; Voors et al., 2012; Dorff, 2017; Oosterhoff et al., 2018; Page, 2018), and even, participation in cultural and artistic activities (Jauk, 2013; Reyes-Martínez et al., 2020). A potential explanation for these behaviors may lie on the social contract theory, which has been helpful explaining political behaviors and other pro-social conducts of victims of crime (Oosterhoff et al., 2018) and disenfranchised populations (Wray-Lake et al., 2018). Under this perspective, it can be suggested that community violence and structural violence (experiences outside the home) could lead to changes in the behaviors of those who reported themselves as victims (see e.g., Oosterhoff et al., 2018; Armesto, 2019). In those individuals, anger or fear could conduct to attend, engage, or consume more cultural or artistic activities, as part of conscious or unconscious strategies to restore their well-being. In the case of domestic violence, a more intimate form of victimization experience, findings could not support their influence into a higher occurrence of cultural participation. This situation suggests a distinctive nature of this type of violence with a potential different treatment or solution, beyond arts and cultural activities.

### 7.2. Conclusion

This manuscript aims to explore the relationship and potential influence of cultural participation on the subjective well-being of those individuals that have experienced victimization, in the context of Mexico. It was guided by interest in understanding alternative solutions for the restoration of the well-being of victims.

In this research, it was possible to answer the main research question (Research Question 1) and support the central hypothesized relationship (Hypothesis 1). It means, it was identified an overall positive influence of the cultural participation activities on the subjective well-being of victims, because, for those who reported higher levels of cultural participation, the probability of a better subjective well-being were higher.

In the case of Research Question 1a and Hypothesis 1a, results partially support them, because not all categories of cultural and artistic activities (e.g., engagement) reported a relationship with subjective well-being dimensions. Contrary, regarding Research Question 1b, scores support them because it was possible to observe that self-perceived victimization lessened the probability of a higher subjective well-being. Concerning Research Question 1c and Hypothesis 1c, findings partially support them due to the lack of association between cultural participation and some specific forms of victimization (e.g., domestic violence). Bearing all this in mind, it is possible to conclude that all these relationships reinforce the idea that individuals potentially coped and adapted to stressful and traumatic situations *via* the cultural participation activities.

In addition, results show most of the expected effects. Namely, most cultural participation variables displayed the proposed effects on the subjective well-being of victims. In other words, they are consistent with most references in the literature. However, considering several aspects of the proposed theoretical model have not been explored before, some unexpected findings arose from this study: a) the null indirect effect of domestic violence *via* cultural attendance, engagement, and consumption to both cognitive well-being and affective balance; and b) the lack of a mediation effect of cultural engagement to affective balance. These findings are not so surprising because, according to theory, subjective well-being, cultural participation, and victimization can be moderated by other factors that were not assessed here. As observed, these moderating factors could explain our distinctive outcomes.

Regarding repercussions, findings may lead to important implications to the design of public policies and interventions and practice, as well as the development of theory and research. In the case of public policy, the evidence here will provide support of the role of cultural and artistic activities as mechanisms of individual and social restoration, in specific, of those who have been victims. It means the need to guarantee the accessibility of cultural services to every population group. In addition, knowledge of the mechanisms that help victims to restore their well-being will be useful to generate programs and interventions related to the attention of victims. As noted, different types of victimization produce distinctive outcomes for individuals, which emphasizes that differentiated treatment is required in every case. To practitioners in the field (e.g., psychologists, social workers, cultural managers) results will fill the gap in the role of cultural and artistic activities as contributors to well-being, physical and mental health, and quality of life. Besides, findings will be helpful in the development of sound theoretical models and methodologies in the field of Victimology. Finally, in terms of research, future investigations will need to take into account the separate and distinctive effects of every type of victimization, under their own circumstances, and the outcomes on the different subjective well-being dimensions. Indeed, the effects of victimization on individuals cannot be considered as monolithic constructs. Researchers must include all these observations toward more effective and accurate solutions to victims and experiences of victimization in Mexico.

### 7.3. Limitations

Some limitations of the research need to be taken into account for the interpretation, discussions, and conclusions of the findings presented here.

A major limitation of this research is the cross-sectional nature of the survey, which does not allow establishing causal relationship between the variables. In addition, since this is a secondary data analysis, other categories of cultural participation (e.g., community celebrations, heritage, traditions, or use of language) or victimization (e.g., secondary or contextual victimization) are not available.

Despite these limitations, this paper reveals an important gap in the attention of victims and the role of cultural and artistic activities in the restoration of well-being.

## Data availability statement

The original contributions presented in the study are included in the article/Supplementary material, further inquiries can be directed to the corresponding author.

## Ethics statement

The studies involving human participants were reviewed and approved by Boston College’s Institutional Review Board approval for analysis of secondary data, under protocol number 20.255.01e.

## Author contributions

JR-M: study conception, design, data analysis, interpretation of results, and draft manuscript. OM-M, ML, and MP-L study conception and critical revision for content. All authors reviewed the results and approved the final version of the manuscript.

## Conflict of interest

The authors declare that the research was conducted in the absence of any commercial or financial relationships that could be construed as a potential conflict of interest.

## Publisher’s note

All claims expressed in this article are solely those of the authors and do not necessarily represent those of their affiliated organizations, or those of the publisher, the editors and the reviewers. Any product that may be evaluated in this article, or claim that may be made by its manufacturer, is not guaranteed or endorsed by the publisher.

## Supplementary material

The Supplementary material for this article can be found online at: https://www.frontiersin.org/articles/10.3389/fpsyg.2022.1082216/full#supplementary-material

## Footnotes

^1^In this research, crime implies violations of law. It usually encompasses the incidence of several types of deviant actions, such as those against life and bodily integrity, personal liberty, liberty and sexual security, heritage, the family, society, and other legal assets (see, e.g., SESNSP, 2020a). Some of these behaviors could be violent, or, cause physical or psychological harm. In international sources, violence is usually only addressed by the number of homicides per one hundred thousand inhabitants because it allows for comparability (see, e.g., OECD, 2011). Besides, victimization refers to an experience of crime and violence, and therefore is accounted as a subjective and personal perspective. It means measures of victimizations usually relies on self-reported perceptions of crime and violent experiences (OECD, 2011). Despite self-reported victimization questions have a high subjective component, they cover the “black number” of crime, i.e., those episodes that are not officially reported. Both, crime and violence produce victims; therefore, the number of victims is interrelated to these phenomena.

^2^Cultural participation refers to how people and groups engage and relate to arts and cultural activities (Schuster, 2007). It includes activities related to cultural and natural heritage, artistic presentations and celebrations, visual arts and crafts, books and press, audiovisual and interactive media, and design and creative services (UNESCO, 2014). In this paper, cultural participation is composed of four general types of practices: attendance, engagement, consumption, and information. Cultural attendance refers to a live attendance of cultural and artistic activities (e.g., going to a concert) (McCarthy and Jinnett, 2001; UNESCO, 2009). Cultural engagement indicates a more active participation than attendance, and even the practice of one artistic activity (e.g., attend a dance workshop) (McCarthy and Jinnett, 2001; NEA, 2009; UNESCO, 2009). Cultural consumption indicates economic transactions and participation through mass media, such as watching TV or attending movies (McCarthy and Jinnett, 2001; NEA, 2009; ESSnet-CULTURE, 2012). And cultural information suggests the searching, communication, diffusion, and repetition of information of cultural and artistic activities through media (McCarthy and Jinnett, 2001; ESSnet-CULTURE, 2012).

^3^Satisfaction with life as a whole, satisfaction with neighborhood/area, satisfaction with standard of living, and satisfaction with safety and security.

^4^Considering we are employing the CFA solution for the measurement model, EFA results are omitted.
