# Supplementary material for: Exploring the influence of cultural participation on the subjective well-being of victims in Mexico
Source: Front Psychol. 2023 Jan 10;13:1082216. doi: 10.3389/fpsyg.2022.1082216 (PMC9871771; doi:10.3389/fpsyg.2022.1082216)
Supplement: Supplementary file 1 [file Data_Sheet_1.docx]

# Appendix A

Table A1. Four-Step Process to Determine a Mediation Relationship

| Step | Analysis | Equation |
| --- | --- | --- |
| 1 | Perform a simple regression analysis with self-perceived victimization (X) to predict subjective well-being (Y) | $Y=b_{0}+ b_{1}X +e$ |
| 2 | Perform a simple regression analysis with self-perceived victimization (X) predicting cultural participation (M) | $M=b_{0}+ b_{1}X +e$ |
| 3 | Perform a simple regression analysis with cultural participation (M) to predict subjective well-being (Y) | $Y=b_{0}+ b_{1}M +e$ |
| 4 | Perform a multiple regression analysis with self-perceived victimization (X) and cultural participation (M) predicting subjective well-being (Y) | $Y=b_{0}+ b_{1}X +b_{2}M+e$ |

*Note*. Adapted from Newsom (n.d.).

Table A2. Descriptive Statistics of Subjective Well-Being Construct, Items

| Interval Variables | N | Mean | SD | Var | Skew | Kurt | Min | Max |
| --- | --- | --- | --- | --- | --- | --- | --- | --- |
| Life satisfaction | 10,654 | 8.05 | 1.89 | 3.57 | -1.33 | 5.20 | 1 | 10 |
| Happiness | 10,654 | 8.37 | 1.74 | 3.06 | -1.68 | 6.72 | 1 | 10 |
| Positive emotions | 10,654 | 7.82 | 2.12 | 4.49 | -1.26 | 4.38 | 1 | 10 |
| Negative Emotions | 10,654 | 3.27 | 2.60 | 6.77 | 0.94 | 2.82 | 1 | 10 |

*Note*. SD) Standard deviation, Var) Variance, Skew) Skewness, Kurt) Kurtosis. Author’s elaboration

Table A3. Descriptive Statistics of Cultural Participation Construct, Items and Indexes

| Categorical Items (N=10,654) | Frequency | Percent |
| --- | --- | --- |
| Attending a concert or a musical show |  |  |
| No | 10,195 | 95.69 |
| Yes | 459 | 4.31 |
| Attending the theater or saw a movie |  |  |
| No | 8,850 | 83.07 |
| Yes | 1,804 | 16.93 |
| Attending a conference, a museum, a gallery, or an exhibition |  |  |
| No | 10,108 | 94.88 |
| Yes | 546 | 5.12 |
| Participating in art classes |  |  |
| No | 10,219 | 95.92 |
| Yes | 435 | 4.08 |
| Participating in craft classes |  |  |
| No | 10,262 | 96.32 |
| Yes | 392 | 3.68 |
| Participating in music-related activities |  |  |
| No | 4,691 | 44.03 |
| Yes | 5,963 | 55.97 |
| Reading books |  |  |
| No | 6,458 | 60.62 |
| Yes | 4,196 | 39.38 |
| Reading articles |  |  |
| No | 5,718 | 53.67 |
| Yes | 4,936 | 46.33 |
| Reading newspapers |  |  |
| No | 5,580 | 52.37 |
| Yes | 5,074 | 47.63 |
| Watching a documentary on TV |  |  |
| No | 6,569 | 61.66 |
| Yes | 4,085 | 38.34 |
| Attendance |  |  |
| No | 8,334 | 78.22 |
| Yes | 2,320 | 21.78 |
| Engagement |  |  |
| No | 9,032 | 84.78 |
| Yes | 1,622 | 15.22 |
| Consumption |  |  |
| No | 2,388 | 22.41 |
| Yes | 8,266 | 77.59 |

*Note*. Author’s elaboration

Table A4. Descriptive Statistics of Self-Perceived Victimization Construct, Items and Indexes

| Categorical Items (N=10,654) | Frequency | Percent |
| --- | --- | --- |
| Physical aggression by someone at home |  |  |
| No | 10,199 | 95.84 |
| Yes | 443 | 4.16 |
| Threats by someone at home |  |  |
| No | 10,389 | 97.67 |
| Yes | 248 | 2.33 |
| Physical aggression by a known person |  |  |
| No | 10,207 | 95.93 |
| Yes | 433 | 4.07 |
| Physical aggression by an unknown person |  |  |
| No | 10,103 | 94.97 |
| Yes | 535 | 5.03 |
| Threats by a known person |  |  |
| No | 10,222 | 96.13 |
| Yes | 412 | 3.87 |
| Threats by an unknown person |  |  |
| No | 9,932 | 93.76 |
| Yes | 661 | 6.24 |
| Discriminatory mistreatment by age |  |  |
| No | 9,861 | 92.56 |
| Yes | 793 | 7.44 |
| Discriminatory mistreatment by skin color or ethnicity |  |  |
| No | 10,375 | 97.38 |
| Yes | 279 | 2.62 |
| Discriminatory mistreatment by disability |  |  |
| No | 10,510 | 98.65 |
| Yes | 144 | 1.35 |
| Discriminatory mistreatment by sex |  |  |
| No | 10,293 | 96.61 |
| Yes | 361 | 3.39 |
| Discriminatory mistreatment by sexual orientation |  |  |
| No | 10,586 | 99.36 |
| Yes | 68 | 0.64 |
| Discriminatory mistreatment for having AIDS |  |  |
| No | 10,641 | 99.88 |
| Yes | 13 | 0.12 |
| Discriminatory mistreatment by religious issues |  |  |
| No | 10,221 | 95.94 |
| Yes | 433 | 4.06 |
| Discriminatory mistreatment by political preferences |  |  |
| No | 10,307 | 96.74 |
| Yes | 347 | 3.26 |
| Discriminatory mistreatment by physical appearance |  |  |
| No | 10,212 | 95.85 |
| Yes | 442 | 4.15 |
| Discriminatory mistreatment by social class |  |  |
| No | 10,014 | 93.99 |
| Yes | 640 | 6.01 |
| Discriminatory mistreatment for being a foreigner |  |  |
| No | 10,607 | 99.56 |
| Yes | 47 | 0.44 |
| Discriminatory mistreatment for other reasons |  |  |
| No | 10,440 | 97.99 |
| Yes | 214 | 2.01 |
| Domestic Violence |  |  |
| No | 10,119 | 95.17 |
| Yes | 513 | 4.83 |
| Community Violence |  |  |
| No | 9,131 | 86.32 |
| Yes | 1,447 | 13.68 |
| Structural Violence |  |  |
| No | 9,005 | 84.52 |
| Yes | 1,649 | 15.48 |

*Note*. Author’s elaboration
